# Is trehalose an autophagic inducer? Unraveling the roles of non-reducing disaccharides on autophagic flux and alpha-synuclein aggregation

**DOI:** 10.1038/cddis.2017.501

**Published:** 2017-10-05

**Authors:** Ye-Seul Yoon, Eun-Duk Cho, Woo Jung Ahn, Kyung Won Lee, Seung-Jae Lee, He-Jin Lee

**Affiliations:** 1 Department of Anatomy, School of Medicine, Konkuk University, Seoul 05029, South Korea; 2 IBST, Konkuk University, Seoul 05029, Korea; 3 Department of Medicine, Seoul National University College of Medicine, Seoul, South Korea; 4 Research Institute of Medical Science, Konkuk University, Seoul 05029, South Korea

## Abstract

Autophagy is a pivotal intracellular process by which cellular macromolecules are degraded upon various stimuli. A failure in the degradation of autophagic substrates such as impaired organelles and protein aggregates leads to their accumulations, which are characteristics of many neurodegenerative diseases. Pharmacological activation of autophagy has thus been considered a prospective therapeutic approach for treating neurodegenerative diseases. Among a number of autophagy-inducing agents, trehalose has received attention for its beneficial effects in different disease models of neurodegeneration. However, how trehalose promotes autophagy has not been fully revealed. We investigated the influence of trehalose and other disaccharides upon autophagic flux and aggregation of *α*-synuclein, a protein linked to Parkinson's disease. In differentiated human neuroblastoma and primary rat cortical neuron culture models, treatment with trehalose and other disaccharides resulted in accumulation of lipidated LC3 (LC3-II), p62, and autophagosomes, whereas it decreased autolysosomes. On the other hand, addition of Bafilomycin A1 to trehalose treatments had relatively marginal effect, an indicative of autophagic flux blockage. In concordance with these results, the cells treated with trehalose exhibited an incremental tendency in *α-*synuclein aggregation. Secretion of *α-*synuclein was also elevated in the culture medium upon trehalose treatment, thereby significantly increasing intercellular transmission of this protein. Despite the substantial increase in *α-*synuclein aggregation, which normally leads to cell death, cell viability was not affected upon treatment with trehalose, suggesting an autophagy-independent protective function of trehalose against protein aggregates. This study demonstrates that, although trehalose has been widely considered an autophagic inducer, it may be actually a potent blocker of the autophagic flux.

Autophagy is an essential cellular process that disposes of dysfunctional or superfluous organelles and proteins through lysosomes.^1^ The degraded products are then released into the cytoplasm to be reused for essential biosynthetic pathways. Major autophagic passages can be further divided into three types: macroautophagy, chaperone-mediated autophagy (CMA), and microautophagy. First, macroautophagy forms a double-membrane phagophore in the cytoplasm that matures into autophagosome and fuses with lysosome to form autolysosome. Second, CMA employs specific lysosomal receptor Lamp2 A to directly translocate unfolded protein across lysosomal membrane with the help of a cytosolic chaperone protein called Heat shock cognate 70.^2^ Lastly, microautophagy involves direct invagination of lysosomal membrane to take up cytoplasmic constituents.

Neurons and other cells afflicted with neurodegenerative disorders display robust accumulation of autophagosomes.^2^ The common neurodegenerative pathology observed is the accumulation of protein aggregates, which may be responsible for cellular dysfunctions such as abnormal synaptic responses and neuronal defects. The deposition of protein aggregates could be caused by disruption of degradation pathways, which accordingly shares linkages to cellular toxicity.^3^ Particularly, mounting evidence suggests that autophagy-lysosomal pathway is a rate-limiting, major pathway involved in the clearance of damaged and aggregated proteins under stress conditions.^4^ Among the research field of Parkinson’s disease (PD), recent progressions have shown that *α*-synuclein, LRRK2, Parkin, PINK1, ATP13A2, Rab7L, and VPS35 are linked to autophagy pathways.^1, 5, 6, 7^

PD, dementia with Lewy bodies, and multiple system atrophy are characterized by abnormal accumulation of *α*-synuclein, an abundant neuronal protein, and thus referred to as synucleinopathies.^8^ The amyloid fibril form of *α-*synuclein is a crucial component of Lewy bodies, the major pathological intracytoplasmic inclusion typically observed in synucleinopathies. *α-*Synuclein consists of 140 amino acids and is a natively unfolded protein that spontaneously develops into amyloid fibrils.^9^ In the neuronal cells of both cellular and animal models, the *α-*synuclein is not only aggregated but also secreted via unconventional exocytosis under various stress conditions.^10, 11, 12^ In PD, Lewy body pathology spreads through a highly specific and predictable pattern in the brain; *α-*synuclein aggregation appears in a few discrete regions of the lower brain stem and the olfactory bulbs, whereas in the later stage it disseminates to larger brain areas.^13^

Inefficient removal of *α*-synuclein through the autophagy-lysosomal pathway has been demonstrated to result in accumulation of the aggregates.^14, 15^ *α-*Synuclein is degraded by both macroautophagy and CMA.^16, 17, 18^

Our previous study has shown that the autophagic dysfunction induced by pharmacological inhibitors or autophagy gene (ATG7) deficiency resulted in the accumulation of *α*-synuclein aggregates in vesicle fractions and increased exocytosis of *α*-synuclein.^12^ Under the same conditions of autophagic dysfunction, we observed augmentations in both transcellular transfer of *α*-synuclein and cell death in the recipient cells.

Approximately a decade ago, disaccharides first received attention as inhibitors of polyglutamine protein aggregation in the cellular model of Huntington disease (HD).^19^ Sugars are vital for the brain functions, where they assist structural development, synaptogenesis, and synaptic transmission by conjugating to proteins and lipids to constitute glycoproteins or glycolipids.^20^ Moreover, they provide cells with energy and are required for neurotransmitter production.^21^

Trehalose (O-*α*,-D-glucopyranosyl-[1→1]-*α*-D-glucopyranoside), a non-reducing disaccharide, has been shown to be neuroprotective in various models of neurodegenerative diseases, such as PD, HD, amyotrophic lateral sclerosis, and tauopathies.^22, 23, 24, 25, 26^ Comprising an *α*, *α*-1,1-glucosidic bond between two *α*-glucose units, trehalose is a naturally occurring sugar in all organisms but for vertebrates.^27^ The advantages of trehalose as a pharmacological agent include high hydrophilicity, chemical stability, and strong resistance to acid hydrolysis and cleavage by glucosidases owing to its non-reducing property. Moreover, in denaturing conditions, trehalose was shown to act as a molecular chaperone to help refold partially denatured proteins.^28, 29^

Currently, the major theory as to how trehalose protects against neurodegeneration in proteinopathy models is that this agent acts as an autophagy inducer. However, the previous studies on trehalose and autophagy rarely investigated the entire process of autophagy, and, thus, the effects of trehalose on autophagic flux remain unknown. Here, we describe a study where the effects of trehalose on autophagic flux and protein aggregate accumulation have been comprehensively examined. Our study provides evidence that trehalose can be a blocker for autophagic flux and that neuroprotective effects of trehalose may be independent of autophagy.

## Results

### Trehalose blocks autophagic flux at the stage of autolysosome formation

**Figure 1:**
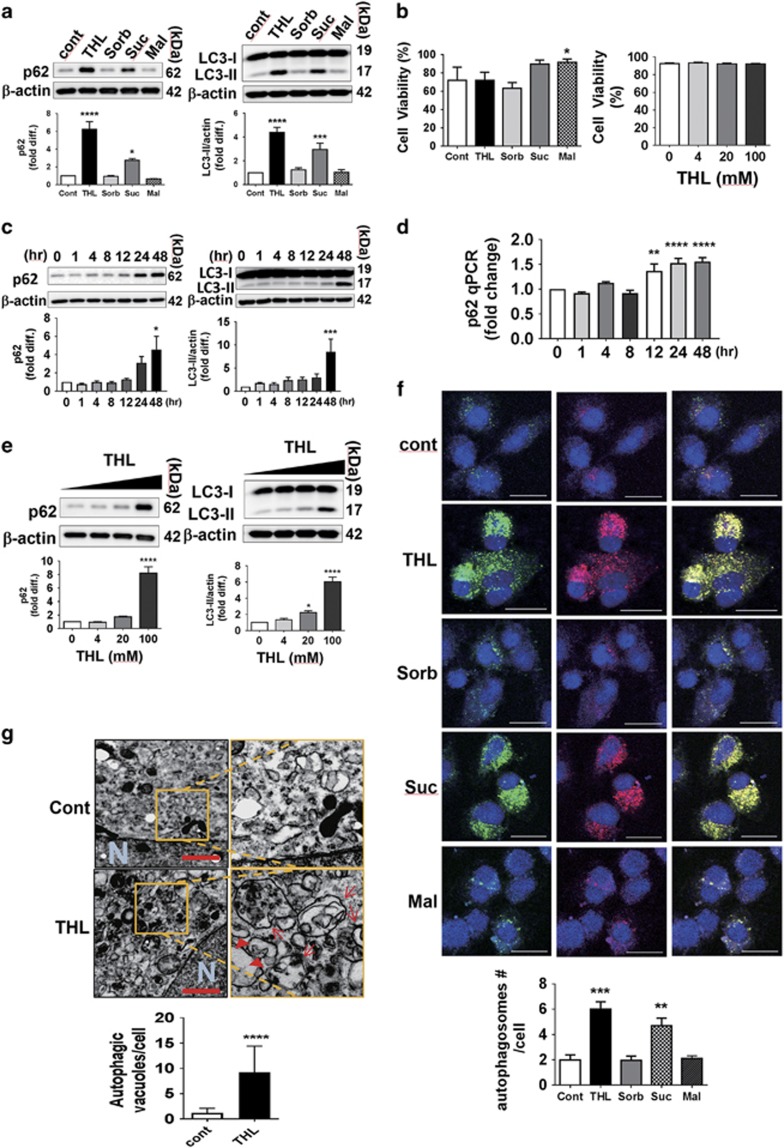
Trehalose increases autophagic markers in SH-SH5Y cells. (**a**) Elevated levels of autophagic substrate p62 and autophagic marker LC3-II in differentiated SH-SY5Y cells treated with disaccharides trehalose (THL) and sucrose (Suc) but not with monosaccharide sorbitol (Sorb) or disaccharide maltose (Mal). (**b**) Viability of cells treated with 100 mM sugars or different doses of trehalose. (**c**) Trehalose increases of p62 and LC3-II proteins in a time-dependent manner. (**d**) Quantitative real-time PCR of p62 in trehalose-treated cells. (**e**) Increasing concentrations of trehalose correlates with levels of p62 and LC3-II proteins. (**f**) Cells expressing tfLC3 (both RFP and GFP tandem repeats fused to LC3) are much more abundant with tfLC3 puncta in non-reducing sugars, such as trehalose and sucrose, than with sorbitol or maltose. (Scale bar: 20 *μ*M). (**g**) Transmission electron microscopy to show increases in number of autophagic vacuoles (arrows) in cells with trehalose treatment compared with controls. The right images are blown-up from the yellow squares in the left image (scale bar: 1 *μ*m)

Differentiated SH-SY5Y cells were treated with two non-reducing disaccharides, trehalose and sucrose, a reducing disaccharide maltose, and a monosaccharide sorbitol at 100 mM concentration for 48 h. Autophagic marker LC3-II and autophagy substrate p62 proteins were then examined (Figure 1a). p62 protein increased more than sixfold in trehalose-treated and threefold in sucrose-treated cells, but were not changed in cells treated with sorbitol or mannose. LC3-II levels also increased significantly in the cells treated with trehalose (fourfold) and sucrose (more than threefold), whereas no changes were observed in cells treated with sorbitol or mannose. Effects of sugars on autophagy were not due to cytotoxicity of these agents (Figure 1b).

Trehalose effect on autophagy was then examined at different times (Figure 1c). The protein levels of p62 and LC3-II started to increase at 24 h and significantly increased to approximately eightfold at 48 h. It has been suggested that autophagy induction transcriptionally activates autophagy genes, such as *p62*, which could be the cause of elevation in the autophagy protein levels.^30, 31^ To examine the effect of trehalose on p62 transcription, real-time quantitative PCR was performed (Figure 1d). There was a slight increase (~1.5-fold) of p62 after 12 h of trehalose incubation. However, p62 transcription increase (~1.5-fold) is much less than what we have observed for p62 protein accumulation (more than eightfold) at 48 h, and knowing that p62 protein half-life is short (~6 h in HeLa cells),^32^ it seems less likely that the p62 transcriptional activation is the major reason for the accumulation of p62 protein in the cell.

The effect of trehalose on autophagy was further confirmed by treating increasing doses of trehalose to cells, from 0 to 100 mM for 48 h, in which dose-dependent elevations of p62 and LC3-II were also observed (Figure 1e). However, trehalose failed to induce phosphorylation of adenosine 5′-monophosphate-activated protein kinase (AMPK), which has been shown to be induced in hepatocyte cultures and activate autophagy (Supplementary Figure 1).^33^

To examine the effect on autophagic flux, we have transduced cells with the recombinant adenoviral vector expressing mRFP-GFP tandem fluorescent-tagged LC3 (tfLC3; adeno/tfLC3).^12^ This approach allowed us to distinguish between autophagosomes (yellow color due to both mRFP and GFP fluorescences) and autolysosomes (red fluorescence due to inactivation of GFP fluorescence in acidic environment of lysosomes; Figure 1f). Addition of trehalose and sucrose increased the number of autophagosomes (yellow) but decreased autolysosomes (red alone). Maltose and sorbitol showed little change in the amounts of both autophagosomes compared with those of the control. These results suggest that the increase of p62 and LC3-II in cells by trehalose and sucrose is due to inhibition of autophagosomes to become autolysosomes. We also confirmed that the number of autophagic vacuoles in cells treated with trehalose increased approximately sevenfold compared with that in control using electron microscopy (Figure 1g).

**Figure 2:**
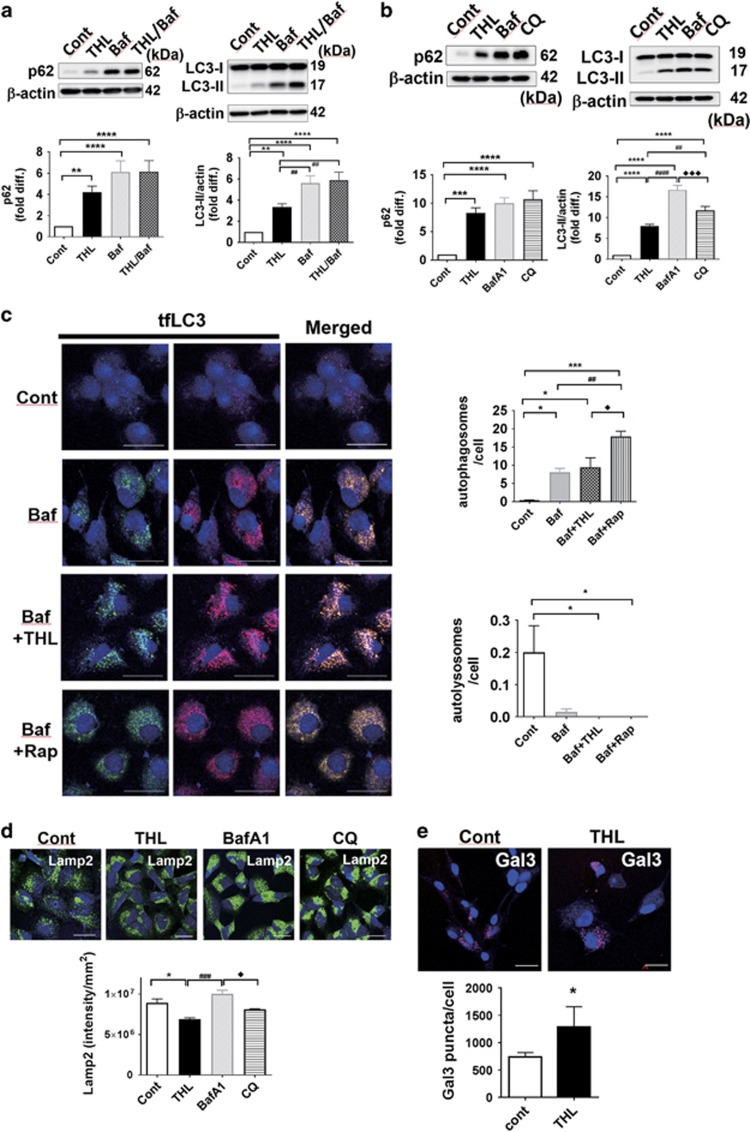
Trehalose (THL) blocks autolysosomal clearance. (**a**) Trehalose (48 h treatment) and BafA1 (Baf; 12 h treatment) increase p62 and LC3-II levels in cells, respectively. (**b**) CQ (24 h), a lysosome-disrupting agent, also increases p62 and LC3-II forms. (**c**) Accumulation of both green and red fluorescences (autophagosomes) and red fluorescence only (autolysosomes) in tfLC3-expressing cells. Note that autophagosomes increased more with Baf+THL compared with Baf+Rap. (**d**) The lysosomal membrane protein LAMP2 levels decreased with THL treatment but not with Baf or CQ. (**e**) Galectin3-mRFP was transfected and Gal3 uptake into lysosomes was observed after digitonin permeabilization. Note that there are more Gal3 puncta with THL treatment compared with control (scale bar: 20 *μ*m)

Autophagic flux can be inhibited when the substrates could not be delivered to lysosomes for degradation. Bafilomycin A1 (BafA1) is a vacuolar-type H^+^ ATPase inhibitor that prevents fusions of autophagosomes and primary lysosomes, thus inhibiting the autophagic flux.^34^ Recently, there has been a concern as to how much time, ranging from minutes to hours, should be adequate enough for a short-term treatment of BafA1 to block fusion.^35^ To circumvent this issue, we treated cells with 50 nM BafA1 for 12 h, which seems to be a sufficient time to block the fusion but 4 h was not (Figure 2a and Supplementary Figure 2). Specifically, differentiated SH-SY5Y cells were treated with trehalose, BafA1, or trehalose and BafA1 together, where trehalose was treated for 48 h and BafA1 was added during the last 12 h before harvesting. The p62 and LC3-II protein levels were verified as shown in Figure 2a. Treatment with trehalose and BafA1 separately resulted in an increase in the levels of both p62 and LC3-II, whereas co-treatment caused the increase in the levels of these proteins to the similar extent as BafA1 treatment alone. These results suggest that trehalose treatment could block the autophagy flux. Comparison with chloroquine (CQ), a lysosomotropic agent that prevents acidification and preventing autophagosome–lysosomal fusion, also showed similar changes in p62 and LC3-II levels, further supporting the results above (Figure 2b).

To examine the effect of trehalose on autophagy initiation, the effects of trehalose were compared with those of rapamycin, a well-known autophagy inducer.^36^ Autophagic degradation was blocked by BafA1 and then trehalose- or autophagy-promoting agent rapamycin was co-treated to measure autophagosome formation. As expected, co-treatment of BafA1 and rapamycin increased the number of autophagosomes but not autolysosomes (Figure 2c). Trehalose and BafA1 together also displayed a slight, but not significant increase in autophagosome numbers compared with BafA1 alone, suggesting that trehalose may not be a potent autophagy inducer and may act more similarly to BafA1 to block the autophagic flux into lysosomes.

Next, we asked whether the autophagy flux inhibition by trehalose resulted from disrupting lysosome integrity. To address this question, we first examined lysosome-associated membrane protein 2 (LAMP2) as a measure of lysosome quantity. LAMP2 protein is a lysosomal integral membrane protein that may engage in the fusion between autophagosomes and lysosomes.^37, 38^ Immunofluorescence staining of LAMP2 showed no significant changes in the LAMP2 levels in the cells treated with BafA1 and CQ, whereas the cells treated with trehalose exhibited a reduction of the LAMP2 staining, possibly indicating decreased lysosome numbers in cells (Figure 2d).

Secondly, we examined the lysosomal membrane integrity by measuring the uptake of fluorescence-labeled galectin3 (Gal3) into lysosomes. Galectins are small, soluble carbohydrate-binding lectins that bind to *β*-galactoside-containing sugars. They are synthesized as cytosolic proteins and are rapidly translocated into lysosomes when the membrane is disrupted and becomes leaky.^39^ mCherry-Gal3 was expressed in cells and Gal3-positive puncta were analyzed (Figure 2e). Trehalose treatment increased Gal3-positive puncta in cells, indicating that lysosomal membrane integrity was compromised. A reduction in lysosome number and the increased lysosomal membrane leakage in trehalose-treated cells suggest that trehalose compromise the lysosomal integrity and function.

**Figure 3:**
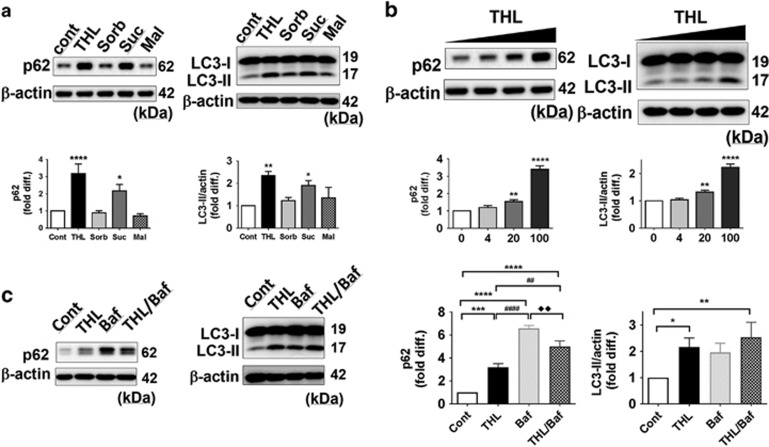
Trehalose increases autophagosomes in rat primary cortical neurons. (**a**) Elevated levels of p62 and LC3-II proteins in cells treated with trehalose (THL) and sucrose (Suc) but not with monosaccharide sorbitol (Sorb) or disaccharide maltose (Mal). (**b**) THL dose-dependent increase of p62 and LC3-II forms in rat neuron culture. (**c**) THL and Baf increased p62 and LC3-II levels in rat neurons similar to SH-SY5Y cells

To confirm these results in primary neurons, rat cortical neurons differentiated for 7 days were treated with 100 mM of trehalose, sorbitol, sucrose, and maltose for 48 h. The results showed similar pattern as in SH-SY5Y cells, with increased protein amount of p62 and LC3-II in trehalose and sucrose-treated cells (Figure 3a). Different doses of trehalose increased the amount of p62 and LC3-II forms in a dose-dependent manner (Figure 3b). Trehalose did not further enhance the effects of BafA1 when co-treated, suggesting that increases of p62 and LC3-II were due to autophagic flux blockade (Figure 3c).

**Figure 4:**
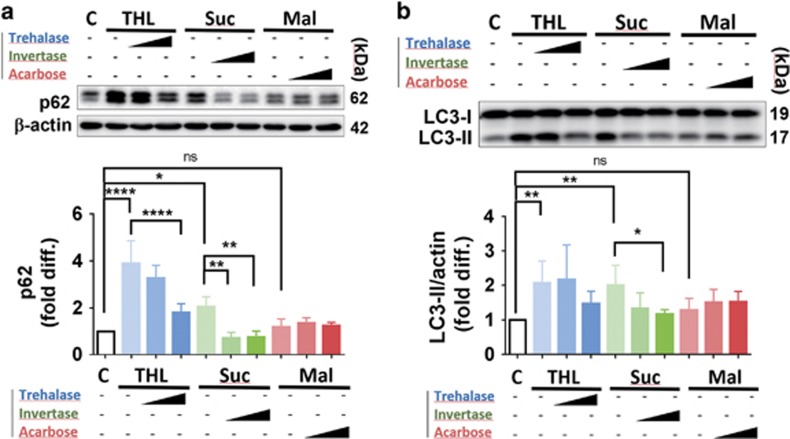
Hydrolysis of disaccharides, THL and Suc, by trehalase and invertase enzymes, respectively, reversed the effects of these disaccharides on p62 (**a**) and LC3-II (**b**) protein levels

To assess whether the effects of trehalose and other disaccharides on autophagy are direct effects of sugar molecules, we utilized hydrolyzing enzymes that break down the disaccharides into monosaccharides (Figure 4). Trehalase is an enzyme that hydrolyzes trehalose into two glucose units. Similarly, invertase hydrolyzes sucrose into glucose and fructose. As these two enzymes are absent in neuronal cells, we exogenously added these enzymes to the culture medium. On the contrary, maltase is present in most cells and, therefore, acarbose, an inhibitor of maltase, was added to prevent breakdown of maltose. The prediction was that addition of trehalase and invertase would decrease the concentrations of trehalose and sucrose, whereas addition of acarbose would increase the concentration of maltose. Our results showed that increasing doses of enzymes, trehalase and invertase, in the culture medium almost negated the effects of trehalose and sucrose on the levels of autophagy markers p62 and LC3-II (Figures 4a and b). Addition of acarbose, in contrast, did not result in significant changes of p62 and LC3-II, possibly due to inefficient internalization of the reagent into cells. Collectively, these results suggest that autophagy flux inhibition is directly exerted by trehalose and sucrose in their disaccharide forms, and is not the secondary effects through hydrolyzed monosaccharides.

### Trehalose induces *α*-synuclein aggregation and cell-to-cell transmission

**Figure 5:**
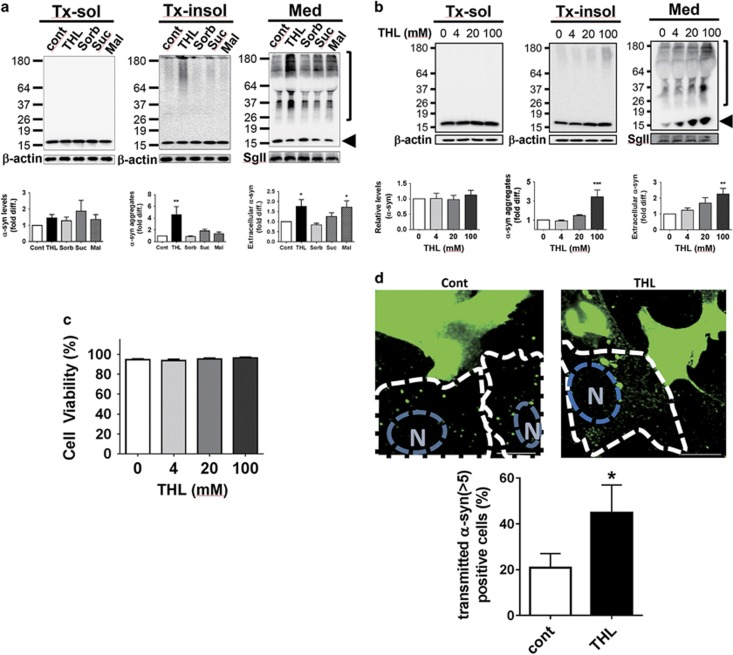
Aggregation and secretion of *α-*synuclein increased with THL treatment. (**a**) Aggregation of *α-*synuclein upon treatment with different sugars. (**b**) Dose-dependent increase of *α*-synuclein aggregates and secretion in THL-treated cells. (arrows: monomer size, bracket: *α-*synuclein aggregates). (**c**) Cell viability of *α-*synuclein-expressing SH-SY5Y cells treated with different doses of THL. (**d**) Cell-to-cell transmission of *α*-synuclein increases with THL treatment. *α-*Synuclein-expressing donor cells (green) were cultured with recipient cells (in dotted lines) and the *α-*synuclein-positive recipient cells were counted

Trehalose has been shown to be protective against neurotoxicity and facilitates the clearance of protein aggregates in HD and PD cell and mouse models.^19, 22, 23, 24, 40^ To determine the effects of trehalose and other sugars on *α-*synuclein aggregation and secretion, differentiated SH-SY5Y cells were transduced with recombinant *α*-synuclein adenoviral vectors (adeno/*α-*syn). Trehalose treatment increased the levels of *α*-synuclein aggregates, whereas other sugars did not (Figure 5a). As for the secretion of *α-*synuclein aggregates, trehalose and maltose led to a significant increase, whereas other sugars had little effects. Enhanced aggregation and secretion of *α*-synuclein correlated with increasing concentrations of trehalose (Figure 5b). Increased *α-*synuclein aggregation and secretion with trehalose treatment could be explained by impaired autophagic flux shown in Figures 1 and 2, which is consistent with our previous results.^12^ *α*-synuclein secretion upon maltose treatment may have different underlying causes, because unlike trehalose, maltose did not have an effect on the autophagic flux.

We then examined cell viability after *α*-synuclein expression and trehalose treatment (Figure 5c). Although trehalose increased *α-*synuclein aggregation, the same treatment did not affect the cell survival. In light of the previous studies, where the levels of *α-*synuclein aggregates strongly correlated with decreased cell viability,^11, 41^ the lack of cell death in trehalose-treated cells with high levels of *α*-synuclein aggregates suggested the protective effects of trehalose against *α-*synuclein aggregates.

In our previous studies, secretion of *α*-synuclein aggregates led to the uptake of this protein into the neighboring cells.^42, 43^ To determine whether trehalose affects cell-to-cell transfer of *α*-synuclein, SH-SY5Y cells expressing *α*-synuclein (donor cells) were co-cultured with naive SH-SY5Y cells (recipient cells). We then measured the percentage of recipient cells with the transferred *α-*synuclein in the cytosol. Our result exhibited a significant increase (approximately twofold) in cell-to-cell transfer of *α-*synuclein in trehalose-treated culture compared with control (Figure 5d). These results support the role of trehalose as an autophagy flux inhibitor, which could interfere with degradation in the lysosomes and lead to accumulation and secretion of *α*-synuclein aggregates.

## Discussion

Autophagic impairment is a common feature often found in many neurodegenerative diseases. The consequent accumulation of various autophagic substrates, damaged organelles, and protein aggregates has been a hitherto meticulously explored topic in neurodegeneration. Recently, trehalose has been considered an autophagy inducer and thus being proposed as a novel therapeutic option.^22, 44^ It has offered a positive direction toward treating several models of neurodegenerative diseases, but failed to provide a precise delineation upon how trehalose drives autophagy. Inconsistent with this general notion, the results of our experiments on disaccharides, including trehalose, demonstrated that they act as strong inhibitors in the overall flow of autophagy. Subsequently, the instability in the autophagy flux by trehalose reflected attenuated membrane integrity of lysosomes and obstructed autophagosome–lysosome fusion. Moreover, the cells treated with trehalose exhibited a remarkable increase in *α*-synuclein protein aggregation and secretion, which represents pathologies of PD and its related diseases.

Cumulative evidence, until recently, has emphasized the neuroprotective effects of trehalose in animal studies as well as in *in vitro* studies.^45^ The oral administration of trehalose for 12 weeks to a HD mouse model displayed improved motor functions, reduced brain atrophy, and less protein aggregates in the brain compared with glucose-fed control mice, indicating that the neuroprotective effect is attributed to trehalose itself and not to elevated glucose levels by trehalose hydrolysis.^46^ Furthermore, in a *Caenorhabditis elegans* model of HD, trehalose extended the mean lifespan and reproductive span while reducing polyglutamine aggregation.^40^ In cell culture models, trehalose cleared A53T mutant *α*-synuclein but not those of the wild type.^24^ Similarly, *α*-synuclein transgenic mice orally fed on trehalose for a week manifested activation of autophagy, increased levels of several chaperone proteins, and suppression of detergent-insoluble *α*-synuclein aggregate formation in their brains.^26^ Another transgenic mouse model expressing mutant SOD1, upon intraperitoneal injections of trehalose, revealed a significantly extended lifespan and a delayed onset and progression of the motor impairments.^25^ In terms of human samples, in the skin fibroblasts from healthy subjects and HD patients, trehalose reversed the effects of proteasome inhibitor epoxomicin and inhibited accumulation of huntingtin proteins in cells.^47^

Although these aforementioned studies highlighted the potentials of trehalose in neuroprotection and autophagy, they had their own limitations in specifically identifying as to which step of autophagy trehalose influences in neuronal cells. In the present study, we demonstrated that, contrary to the current views in the field, trehalose inhibits autophagy flux, leading to the accumulation of enlarged autophagosomes in cells. Although to a less extent, sucrose resulted in a similar outcome, whereas monosaccharide sorbitol and disaccharide maltose, which contain endogenous hydrolyzing enzymes, were devoid of such effect. The deposition of LC3-II and p62 in autophagosomes suggests that autophagy flux may not function properly, and that the newly formed autophagosomes could not reach lysosomes for their degradation (Figure 1). In fact, the cells co-treated with trehalose and BafA1, an inhibitor of autophagosome–lysosome fusion, had no additive effect compared with those treated with BafA1 or trehalose alone, further demonstrating that the overall autophagic flow was obstructed (Figure 2).

Moreover, the addition of hydrolyzing enzymes mitigated the effects of trehalose and sucrose on autophagy as these sugars were broken down to monosaccharides. A recent report also showed that sucrose, in addition to trehalose and raffinose, which do not normally possess endogenous hydrolyzing enzymes, caused the intracellular accumulations of LC3-II and enlarged vesicle accumulations, whereas the cells supplemented with hydrolyzing enzymes showed reversion of the effects.^48^

A detailed account of how trehalose and other disaccharides could block autophagy flux still remains unanswered. However, a previous study had observed osmotic enlargement of mature lysosomes in cells treated with sucrose, indicating that disrupted lysosomes may be the underlying reason for inhibition of autophagosome–lysosome fusion.^49^ In agreement with these findings, our results (Figures 2d and e) also depicted that the membrane integrity of lysosomes was compromised. On the basis of these results, we speculate that trehalose and other disaccharides may disturb lysosome integrity and its function, which could subsequently hinder the lysosomal fusion with autophagosomes.

In a recent report, trehalose inhibited solute carrier 2 A family (or glucose transporter/GLUT family) proteins that impeded with uptake of glucose and other monosaccharides into the hepatic cells. This led to deprivation of glucose that led to AMPK-dependent activation of autophagy.^33^ Another recent work had shown SLC2A8 (GLUT8) to be a mammalian trehalose transporter in hepatocytes.^50^ SLC2A8 was required to induce autophagy in an AMPK-dependent manner.

However, as these authors have mentioned, LC3-II accumulation was not SLC2A8-dependent in N2A neuroblastoma cells.^50^ In fact, SLC2A8 is not localized in the plasma membrane of neurons. Therefore, the effect of trehalose on SLC2A and AMPK might be tissue-specific and may not occur ubiquitously. This could explain why we do not see activation of AMPK by trehalose treatment and the levels of autophagic marker proteins change much slowly than in hepatocytes. Nevertheless, further studies are needed for determining the definite mechanism behind these observations.

Our result of an increase in *α*-synuclein aggregates with trehalose is somewhat contradictory to the previous reports.^19, 24^ However, these previous studies had limitations in that they displayed changes in only the monomeric *α*-synuclein levels and not those in the SDS-resistant high MW aggregates. Trehalose has been reported to stabilize proteins in the native state during heat shock and to reduce aggregation in yeast models.^28^ Interestingly, trehalose was rapidly broken down afterwards. When hydrolyzing enzyme trehalase degradation was inhibited, the reactivation of denatured proteins by other molecular chaperones was hampered. Thus, although the effects of trehalose on the autophagic flux may explain the increase in *α*-synuclein aggregation, modulation of protein folding may also explain as to why *α*-synuclein aggregation was increased in neuronal cells.

Another noteworthy observation was that even though the cells treated with trehalose exhibited augmented *α*-synuclein aggregate accumulation and secretion, the cell viability of these cells did not change (Figure 5c).^14^ The cause for this dissociation between protein aggregation and cell death remains unclear. The explanation for the dissociation could include the possibility that the water-absorbing and protein-stabilizing properties of trehalose may have interfered with the toxic effects of protein aggregates by separating them away from other cellular components. There have been many studies showing that trehalose and other disaccharides or oligosaccharides have cytoprotective effects.^45^ Many of these studies suggested that autophagy activation may be responsible for the cell defense. However, given our current results that trehalose could act as a blocker of autophagic clearance, its protective effects may be independent from the autophagy pathway.

We described new functions of trehalose and other disaccharides as the blockers of autophagic pathway. Trehalose caused impairment of the lysosomal membrane integrity, which led to the inhibition of autophagosome–lysosome fusion. Furthermore, it induced aggregation of *α*-synuclein in neuronal cells. Despite the increased aggregation, cell viability did not change upon trehalose treatment. Therefore, trehalose may be neuroprotective as other studies concluded previously, but through a mechanism independent of autophagy induction.

In conclusion, our results collectively raise a question on the commonly accepted notion that trehalose is an autophagy inducer and instead suggest that it is rather an autophagic flux blocker. Despite being an autophagy blocker, trehalose still exhibits neuroprotective effects, perhaps through an autophagy-independent mechanism.

## Materials and methods

### Reagents and antibodies

Primary antibodies used are as follows: *α*-synuclein monoclonal antibodies Ab274 and Ab62 from our laboratory,^51^ *α*-synuclein, AMPK, p-AMPK, and p62 antibodies (Cell Signaling Technology, Danvers, MA, USA), p62 (BD Biosciences, San Jose, CA, USA), secretogranin II (Santa Cruz Biotechnology Inc., Santa Cruz, CA, USA), LC3B antibody (Abcam, Cambridge, UK), and *β*-actin antibody (Sigma-Aldrich Corp., St. Louis, MO, USA). A monoclonal antibody for LAMP2 (H4B4) developed by Drs August and Hildreth was obtained from the Developmental Studies Hybridoma Bank developed under the auspices of the NICHD, National Institutes of Health and maintained by the University of Iowa, Department of Biological Sciences, Iowa City, IA, USA.

Retinoic acid, poly-L-lysine, trehalose, sorbitol, sucrose, maltose, rapamycin, CQ, trehalase, invertase, and acarbose were also purchased from Sigma. BafA1 was purchased from EMD Millipore (Billerica, MA, USA). pLVX-mCherry-Gal3 plasmid was a kind gift from Dr. Edward M Campbell.^52^

### Animal use

All animal uses were approved by the Konkuk University Institutional Animal Care and Use Committee (KU IACUC). Pregnant Sprague–Dawley rats (E16) were purchased from Orient Bio Inc., Seongnam, Korea. Purchased pregnant rats were killed by asphyxiation in CO_2_ chamber and the embryos were removed immediately.

### Cell culture

The human neuroblastoma cell line, SH-SY5Y, was maintained and differentiated as described previously.^14^ For culture of rat primary cortical neurons, pregnant E16 Sprague–Dawley rats were dissected and cultured as described previously.^14^

### *α*-Synuclein, tfLC3, mCherry-Gal3 expression

Differentiated SH-SY5Y cells were transduced with recombinant adenoviral vectors (adeno/*α*-syn or adeno/tfLC3) as previously described.^12, 14^ Briefly, cells were transduced at a multiplicity of infection of 33 in a half volume of fresh growth medium. Following 90 min of incubation, the remaining half of fresh growth medium was added, and the cells were incubated overnight. The next day, the medium was replaced with fresh medium and incubated further before treatment.

For counting autophagosomes and autolysosomes after tfLC3 expression, puncta that shows fluorescence intensity >10 000 were counted.

pLVX-mCherry-Gal3 plasmid was electroporated into SH-SY5Y cells for expression.

### Sugar supplements

Growth medium with 100 mM or as indicated amount of trehalose, sorbitol, sucrose, or maltose was freshly prepared and was added to the differentiated SH-SY5Y cells transduced with adeno/*α*-syn on day 2. Cells were further maintained for 48 h or as indicated.

### Treatment of autophagy effectors

Autophagy inhibitors, BafA1 and CQ, or autophagy activator, rapamycin, were used as described.^12^ Briefly, differentiated SH-SY5Y cells and rat primary neurons, with or without expressing *α*-synuclein or tfLC3, were treated with 50 nM BafA1, 125 *μ*M CQ, or 100 nM rapamycin in growth medium on day 6 of differentiation for SH-SY5Y and on day 7 for rat cortical neurons. For double treatment of BafA1 and rapamycin or trehalose, differentiated SH-SY5Y cells were pretreated with 50 nM BafA1 for 30 min before the medium was changed with 50 nM BafA1 co-treated with 100 nM rapamycin or 100 mM THL. Cells were further incubated for 12 h or as indicated.

### Treatment of digestive enzymes of sugar

Trehalase (final ~0.00125 U, ~0.005 U; 1 U=amount of enzyme to hydrolyze 1 *μ*mole of trehalose into 2 *μ*mole of glucose in 1 min at pH 5.7, 37 ^o^C; Sigma T8778-5UN), invertase (final 6.8 *μ*g/ml, 34 *μ*g/ml; Sigma I4504), and acarbose (final 0.64 *μ*g/ml, 3.2 *μ*g/ml; Sigma A8980) were treated on day-7 cultured rat primary neurons. Cells were maintained further for 48 h.

### Co-culture experiments

For mixed culture of *α*-synuclein-expressing cells with wild-type cells, differentiated SH-SY5Y cells transduced with recombinant adeno/*α*-syn (donor cells; day 1 of infection) were added to differentiated SH-SY5Y cells (acceptor cells) and cultured for 72 h.

### Preparation of cell extracts and conditioned medium

Cell extracts were obtained as described previously.^53^ Briefly, cells were rinsed with PBS and ice-cold extraction buffer (PBS/1% Triton X-100/protease inhibitor cocktail) was added before the cells were collected with cell scraper. After incubation on ice for 10 min, the cell extract was centrifuged at 16 000 × *g* for 10 min, and the supernatant and the pellets were separated for further analysis. The conditioned medium was centrifuged at 1000 × *g* for 10 min and the transferred supernatant was spun again at 10 000 × *g* for 20 min. The recovered supernatant was stored with protease inhibitor cocktail at −80 ^o^C until further analysis.

### Western blotting

Western blotting was performed as described previously.^53^ Chemiluminescence detection was performed using the FUJIFILM Luminescent Image Analyzer LAS-3000 and Multi Gauge (v3.0) software (FUJIFILM, Tokyo, Japan).

### Cell viability assay

Cells were trypsinized and divided into two tubes. Accustain solution with detergent (Sigma) was added to one of the tubes to permeabilize cells. Accustain solution (without detergent) was then added to both tubes to label total cells (detergent-added cells) and damaged cells (no detergent). Cells from both tubes were counted by ADAM cell counter (NanoEnTek, Seoul, Korea).

### Digitonin permeabilization of cells

To observe the membrane-bound LC3 in tfLC3-overexpressing cells, cells were permeabilized with digitonin as described.^54^ Briefly, cells were rinsed in Hank’s balanced salt solution twice and incubated with 30 *μ*M digitonin at RT for 5 min. Cells were washed with Hank’s balanced salt solution and then fixed in 4% paraformaldehyde for further staining.

### RNA extraction and quantitative real-time PCR

Total RNA was extracted using RNeasy Mini Kit from Qiagen (Hilden, Germany), and was reverse-transcribed using a High Capacity cDNA reverse transcription kit from Applied Biosystems (Foster City, CA, USA). Quantitative real-time PCR was performed on a LightCycler 480 II using the LightCycler 480 SYBR Green I Master (both from Roche, Basel, Switzerland), as recommended.

Primers used are as below:

p62/SQSTM1 Forward: 5′-AAGAACGTTGGGGAGAGTGT-3′, p62/SQSTM1 Reverse: 5′-CTGTGCTGGAACTCTCTGGA-3′ and GAPDH Forward: 5′-GAGTCAACGGATTTGGTCGT-3′, GAPDH Reverse: 5′-TGGAAGATGGTGATGGGATT-3′.

### Electron microscopy

Cells were grown in 100-mm dishes and fixed in the Karnovsky’s fixation solution (2% glutaraldehyde, 2% paraformaldehyde, 0.5% CaCl_2_). After immersing in 1% osmium tetroxide for 1.5 h, cells were dehydrated in 50, 60, 70, 80, 90, 95, and 100% of absolute ethanol. Cells were infiltrated with propylene oxide and EPON mixture (EPON 812, MNA, DDSA, DMP30) for 10 min before being embedded in the EPON mixture. After embedding, the cells were sectioned with LEICA EM UC-7 Ultra-microtome (Leica Microsystems, Wetzlar, Germany), and then stained with 6% uranyl acetate and lead citrate. The grids were observed using transmission electron microscopy JEM-1011 (JEOL, Tokyo, Japan) and analyzed using Megaview III software (Soft Imaging System, Germany). Fifty cells from control and trehalose-treated each were counted for autophagic vacuoles. Puncta with >1.5 *μ*m and the fluorescence intensity >12 800 were counted per cell.

### Immunofluorescence cell staining

The cell-staining procedure has been described elsewhere.^53^ Briefly, cells grown on poly-L-lysine-coated coverslips were fixed in 4% paraformaldehyde in PBS. The fixed cells were permeabilized with 0.1% Triton X-100 before being incubated in blocking solution (5% bovine serum albumin and 3% goat serum in PBS). Primary antibodies were diluted in blocking solution and added to the cells. After being washed in PBS, the cells were incubated with fluorescent dye Alexa488, Cy2, Rhodamine red-X, or Alexa647-conjugated secondary antibodies (Jackson Immunoresearch Laboratories, PA, USA) and then washed again in PBS. The nuclei were stained with TOPRO-3 dye (ThermoFisher, MA, USA), and the coverslips were mounted on slides in Antifade reagent (ThermoFisher). The cells were observed under an Olympus FV1000 confocal laser-scanning microscope.

### Statistical analysis

All experiments were repeated at least three or four times. The values in the figures are expressed as the mean±S.E.M. Null hypotheses of no difference were rejected if *P*-values were less than 0.05. The graphs were drawn and the data were analyzed by means of one-way ANOVA with Dunnett’s or Tukey’s post-test with Prism 7 software (Graphpad Software Inc., CA, USA. *P<0.05, **P<0.01, ***P<0.001, ****P<0.0001.

## Publisher’s Note

Springer Nature remains neutral with regard to jurisdictional claims in published maps and institutional affiliations.
