# Supplementary material for: Is trehalose an autophagic inducer? Unraveling the roles of non-reducing disaccharides on autophagic flux and alpha-synuclein aggregation
Source: Cell Death Dis. 2017 Oct 5;8(10):e3091–. doi: 10.1038/cddis.2017.501 (PMC5682667; doi:10.1038/cddis.2017.501)
Supplement: Supplementary Figures [file cddis2017501x1.pdf]

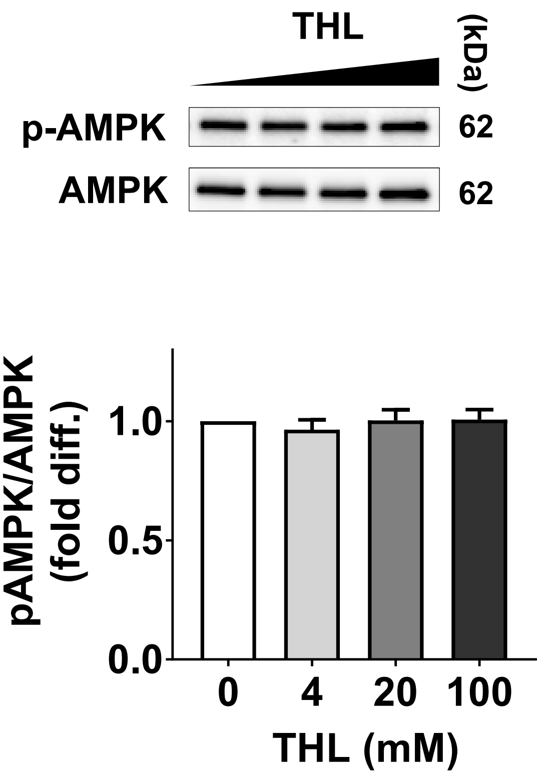

Suppl. Fig. 1 Trehalose do not activate AMPK pathway. Increasing concentrations of trehalose were treated and phosphorylation of AMPK was compared.

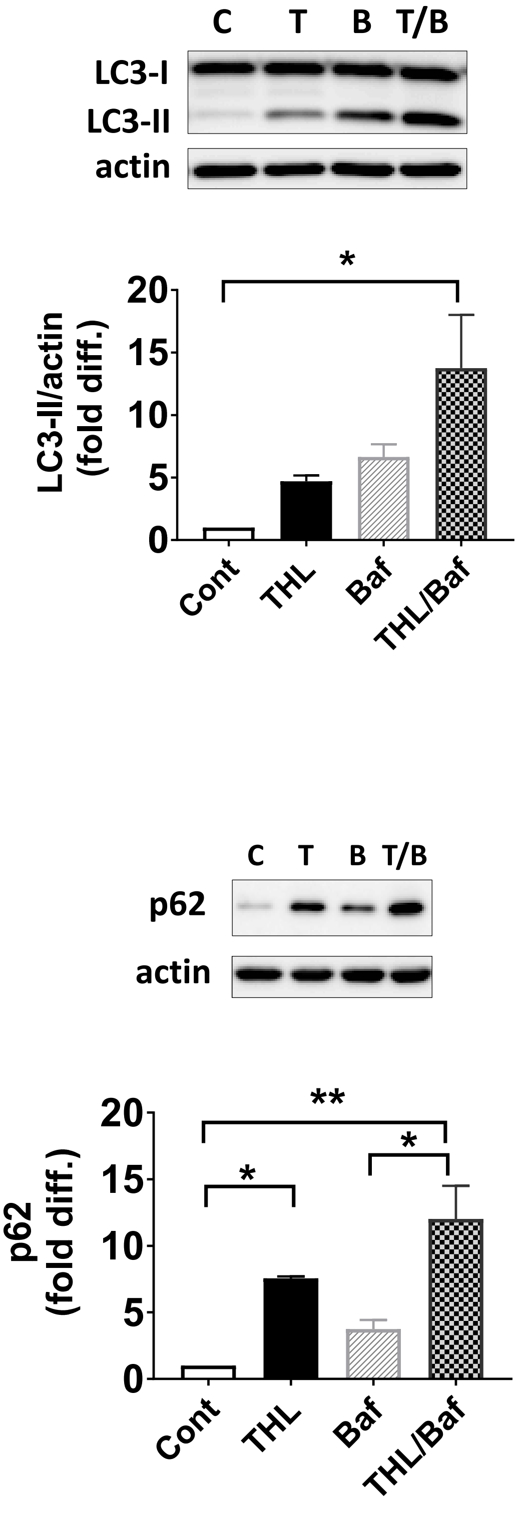

Suppl. Fig. 2 Trehalose (48h) and BafA1(4h) increase p62 and LC3-II protein levels in SH-SY5Ycells. Note that 4 h BafA1 treatment showed much weaker effect in elevating levels of p62 and LC3-II than what was observed with 12h treatment (Fig. 2A).
